# Supplementary material for: A General Analytic Approach to Predicting the Best Antibiotic Dosing Regimen
Source: bioRxiv. 2025 Sep 17:2025.09.13.676026. Preprint. [Version 1] doi: 10.1101/2025.09.13.676026 (PMC12458139; doi:10.1101/2025.09.13.676026)
Supplement: Supplement 1 [file media-1.pdf]

Supplementary Material: A General Analytic Approach to Predicting the Best Antibiotic Dosing Regimen

Leah Childers[1], Pia Abel zur Wiesch[2], and Jessica M. Conway[1]

[1]Department of Mathematics, Pennsylvania State University, University Park PA, U.S.A.
[2]Department of Pharmacy, UiT – The Arctic University of Norway, Tromsø, Norway

Contents

A Definition of Parameters **2**

B Proof of Preliminary Concavity Lemmas **2**

C Proof of Theorem 3 **4**

D Completed Details for Numerical Simulations **6**

D.1 Dose Response Curves of Rifampin, Ciprofloxacin, Streptomycin, and Tetracycline . . . 6

D.2 Full Regimen Details for Ciprofloxacin and Rifampin Simulations 6

E Holding Total Antibiotic Amount Constant **6**

F Code for Figures and Numerical Demonstrations **9**

A Definition of Parameters

Table A1 contains all the parameters we use and reference in the paper in the order they appear.

**Parameter**	**Definition**
T	Single dose treatment length (h)
T_p	Pulse length (for step model) (h)
AUC	Area under the curve of the antibiotic concentration (mg · h/L)
D_1	Constant-concentration dose level (mg/L)
D_2	Pulse (step) dose level (mg/L)
n	Hill coefficient (steepness) of Hill function (unitless). Regoes et. al uses κ [3], but we use n throughout
$G_{\max}$	Maximum growth rate of bacteria (h^{-1}), also called $\psi_{\max}$ in Regoes et al. [3]
t_{half}	Half-life of the antibiotic (h)
λ	Exponential decay constant ($\lambda = \ln(2)/t_{half}$) ($h^{-1}$)
A_0	Initial drug concentration (mg/L)
$\psi_{\min}$	Minimum growth rate of bacteria (h^{-1})
MIC	Minimum inhibitory concentration of the antibiotic (mg/L)
V	Volume of distribution of the antibiotic (L)

Table A1: List of parameters referenced throughout, in order of appearance.

B Proof of Preliminary Concavity Lemmas

Lemma 1. *Consider a function $g : [0, \infty) \to \mathbb{R}$.*

1. *If g is strictly concave down and $g(0) = 0$, then $\frac{1}{x}g(x)$ is strictly decreasing.*
2. *If g is strictly concave up and $g(0) = 0$, then $\frac{1}{x}g(x)$ is strictly increasing.*

Proof.

1. By the definition of strictly concave down, $\forall w \neq z \in [0, \infty)$ and $\alpha \in (0, 1)$, we have

$$(1 - \alpha)g(z) + \alpha g(w) < g((1 - \alpha)z + \alpha w).$$

Fix $x < y \in (0, \infty)$. Note $\frac{x}{y} \in (0, 1)$. So the inequality remains true for $z = 0$, $w = y$, and

$\alpha = \frac{x}{y}$, which, since $g(0) = 0$, gives us

$$\frac{x}{y} g(y) < g\left(\frac{x}{y} y\right)$$
$$\frac{1}{y} g(y) < \frac{1}{x} g(x)$$

as needed.

2. By the definition of strictly concave up, $\forall w \neq z \in [0, \infty)$ and $\alpha \in (0, 1)$, we have

$$(1 - \alpha) g(z) + \alpha g(w) > g((1 - \alpha) z + \alpha w).$$

Fix $x < y \in (0, \infty)$. Note $\frac{x}{y} \in (0, 1)$. So the inequality remains true for $z = 0$, $w = y$, and $\alpha = \frac{x}{y}$, which, since $g(0) = 0$, gives us

$$\frac{x}{y} g(y) > g\left(\frac{x}{y} y\right)$$
$$\frac{1}{y} g(y) > \frac{1}{x} g(x)$$

as needed. □

Lemma 2. *Given three non-colinear points $(x_1, y_1), (x_2, y_2), (x_3, y_3) \in \mathbb{R}^2$, we can draw either a concave up or a concave down curve through all three points, but not both.*

Proof. Fix three non-colinear points $(x_1, y_1), (x_2, y_2), (x_3, y_3) \in \mathbb{R}$ and assume $x_1 < x_2 < x_3$. Define

$$m_1 = \frac{y_2 - y_1}{x_2 - x_1}, \quad m_2 = \frac{y_3 - y_2}{x_3 - x_2}.$$

Now define

$$f(x) = \begin{cases} m_1(x - x_2) + y_2, & x \leqslant x_2 \\ m_2(x - x_2) + y_2, & x \geqslant x_2. \end{cases}$$

Note $f(x_i) = y_i$ for $i = 1, 2, 3$. Since the points are not colinear, $m_1 \neq m_2$, so exactly one must be true:

1. If $m_1 > m_2$, then f is concave down.
2. If $m_1 < m_2$, then f is concave up.

Now, WLOG, assume $m_1 > m_2$. We claim that no concave up curve can be drawn through the three points. Consider

$$g(x) = \frac{y_3 - y_1}{x_3 - x_1}(x - x_1) + y_1$$

which is the line that passes through (x_1, y_1) and (x_3, y_3). Note $x_1 < x_2 < x_3$ and $g(x_2) < y_2$, so any curve which passes through all three points will contradict the definition of concave up. □

C Proof of Theorem 3

Theorem 3. *Given a constant AUC and dose interval T, the following hold true when comparing drug regimens in the decay model:*

i. If the dose response curve is concave up, then the CC regimen performs better than the periodic regimen.

ii. If the dose response curve is concave down, then the periodic regimen performs better than the CC regimen.

Proof. Fix initial concentration A_0, treatment length T, and half-life t_{half} for the periodic regimen as defined in Section 2.3. Then the AUC is $\frac{A_0}{\lambda}(1-e^{-\lambda T})$, so the CC regimen with the same AUC has drug concentration $D_C = \frac{A_0}{\lambda T}(1-e^{-\lambda T})$ given over the interval $[0, T]$. For later, also note that the interval of antibiotic concentration for $[0, T]$ of the periodic regimen is $[A_0 e^{-\lambda T}, A_0]$ and $A_0 e^{-\lambda T} < D_C < A_0$ because $TA_0 e^{-\lambda T} < \int_0^T A(t)dt < TA_0$.

As before, we consider a general PD model

$$\frac{db}{dt} = R\left(f(t)\right) \cdot b(t)$$

where R is the dose response curve and b is the bacteria population. Recall our previous notation $B(t) := \ln\left(b(t)\right)$. Then our antibiotic concentration functions, A_1 and A_2 for constant and periodic regimens respectively, are given by

$$A_1(t) = \frac{A_0}{\lambda T}(1-e^{-\lambda T}), \quad A_2(t) = A_0 e^{-\lambda t}$$

on $[0, T]$ and we have

$$\begin{aligned} B_1'(t) &= R\left(A_1(t)\right) = R\left(A_0 e^{-\lambda t}\right) \\ B_2'(t) &= R\left(A_2(t)\right) = R\left(\frac{A_0}{\lambda T}(1-e^{-\lambda T})\right). \end{aligned}$$

We will assume WLOG $B_1(0) = B_2(0) = 1$. We will again only prove the result for concave up R; the result for concave down R follows by flipping all of the inequalities. Since R is concave up, $R(x) = R_0 - \tilde{R}(x)$ where $\tilde{R}$ is concave down and $\tilde{R}(0) = 0$. We begin with the following equivalent statements:

$$\begin{aligned} B_2(T) - B_1(T) &> 0 \\ 1 + \int_0^T R\left(A_0 e^{-\lambda t}\right) dt - \left(1 + \int_0^T R\left(\frac{A_0}{\lambda T}\left(1-e^{-\lambda T}\right)\right) dt\right) &> 0 \\ \int_0^T R\left(A_0 e^{-\lambda t}\right) dt - TR\left(\frac{A_0}{\lambda T}\left(1-e^{-\lambda T}\right)\right) &> 0 \end{aligned}$$

For the first term, we apply the change of variables $u = \lambda T e^{-\lambda t}$ to get

$$\begin{aligned}
\int_0^T R\left(A_0 e^{-\lambda t}\right) dt &= -\frac{1}{\lambda}\int_a^b \frac{R\left(\frac{A_0}{\lambda T}u\right)}{u} du \\
&= -\frac{1}{\lambda}\int_a^b \frac{R_0 - \tilde{R}\left(\frac{A_0}{\lambda T}u\right)}{u} du \\
&= -\frac{R_0}{\lambda}\left(\ln(b) - \ln(a)\right) + \frac{1}{\lambda}\int_a^b \frac{\tilde{R}\left(\frac{A_0}{\lambda T}u\right)}{u} du \\
&= R_0 T + \frac{1}{\lambda}\int_a^b \frac{\tilde{R}\left(\frac{A_0}{\lambda T}u\right)}{u} du
\end{aligned} \tag{C1}$$

where

$$a = \lambda T, \quad b = \lambda T e^{-\lambda T}.$$

For the second term, we have

$$-TR\left(\frac{A_0}{\lambda T}\left(1 - e^{-\lambda T}\right)\right) = -R_0 T + \tilde{R}\left(\frac{A_0}{\lambda T}\left(1 - e^{-\lambda T}\right)\right) T. \tag{C2}$$

We now use $\tilde{R}(0) = 0$, $0 < e^{-\lambda T} < 1$, and the definition of $\tilde{R}$ concave down to get

$$T\tilde{R}\left(\left(1 - e^{-\lambda T}\right)\frac{A_0}{\lambda T}\right) \geqslant T\left(1 - e^{-\lambda T}\right)\tilde{R}\left(\frac{A_0}{\lambda T}\right). \tag{C3}$$

Combining eq.(C1), (C2), and (C3), it suffices to show

$$\begin{aligned}
\frac{1}{\lambda T}\int_a^b \tilde{R}\left(\frac{A_0}{\lambda T}u\right) du + \left(1 - e^{-\lambda T}\right)\tilde{R}\left(\frac{A_0}{\lambda T}\right) &> 0 \\
\frac{(b-a)}{1 - e^{-\lambda T}}\frac{1}{\lambda T}\int_a^b \frac{\tilde{R}\left(\frac{A_0}{\lambda T}u\right)}{u} du + (b-a)\tilde{R}\left(\frac{A_0}{\lambda T}\right) &> 0 \\
\int_b^a \frac{\tilde{R}\left(\frac{A_0}{\lambda T}u\right) - u\tilde{R}\left(\frac{A_0}{\lambda T}\right)}{u} du &> 0.
\end{aligned}$$

Now, since $\frac{1}{u} > 0$ for $u \in \left[\lambda T e^{-\lambda T}, \lambda T\right]$, it suffices to show

$$\tilde{R}\left(\frac{A_0}{\lambda T}u\right) > u\tilde{R}\left(\frac{A_0}{\lambda T}\right).$$

We prove this with the definition of $\tilde{R}$ concave down. If $0 < u \leqslant 1$, the identity is trivial. If $u > 1$, construct $\{u_i\}_{i=1}^n$ such that $\sum_{i=1}^n u_i = u$ and each $u_i \in (0,1)$. The by

subadditivity of concave down functions, we have

$$\begin{aligned}\tilde{R}\left(\frac{A_0}{\lambda T}u\right) = \tilde{R}\left(\frac{A_0}{\lambda T}\sum_{i=1}^{n}u_i\right) &\geqslant \sum_{i=1}^{n}\tilde{R}\left(\frac{A_0}{\lambda T}u_i\right)\\ &> \sum_{i=1}^{n}u_i\tilde{R}\left(\frac{A_0}{\lambda T}\right)\\ &= u\tilde{R}\left(\frac{A_0}{\lambda T}\right)\end{aligned}$$

as needed. □

D Completed Details for Numerical Simulations

D.1 Dose Response Curves of Rifampin, Ciprofloxacin, Streptomycin, and Tetracycline

Figure D1 shows the dose response curves of rifampin, ciprofloxacin, streptomycin, and tetracycline against *E. coli* in vitro. The dose response curves are parametrized from data in [3] and are on a $\log(x)$ scale. Note this means that for the antbiotics with $n > 1$, the inflection point does not appear at the graph's visual inflection point. A comparison we made previously was that the inflection point of ciprofloxacin is much lower than the inflection point of rifampin, which is clear from this figure.

D.2 Full Regimen Details for Ciprofloxacin and Rifampin Simulations

Table D2 and Table D3 contain the full details of the dosing regimens we defined in Section 3.2 as well as each drug's PD parameters.

E Holding Total Antibiotic Amount Constant

Throughout this paper, we have assumed that the AUC of the drug concentration function of each regimen is constant between the regimens we compare. This means that the total amount of antibiotic administered in each regimen may be different, especially when we consider accumulation and decay of the drug. However, in practice, optimizing the dosing regimen holding constant the total amount of antibiotic used may be desired, such as when dealing with a limited drug supply. The analytic results for this case are not as straightforward as the results we derived in this paper, but we can still make some observations.

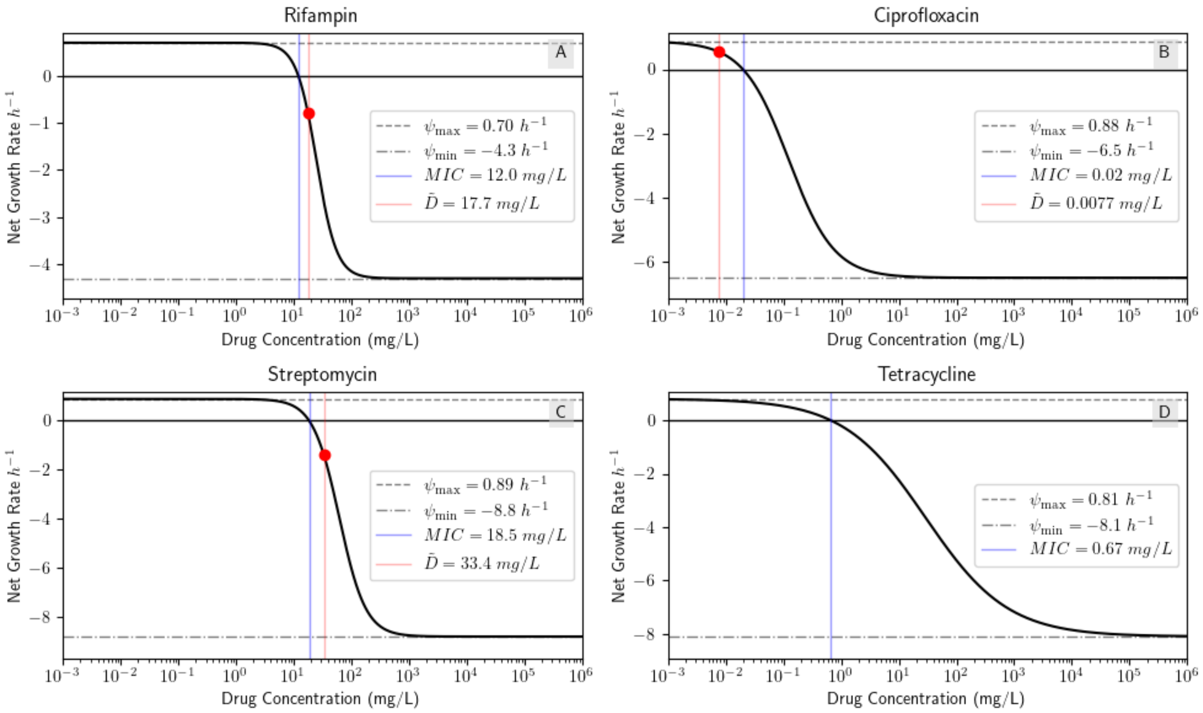

Figure D1: Dose response curves of rifampin, ciprofloxacin, streptomycin, and tetracycline. Data from [3].

When we explored the decay model, the function $f(t)$ always represented the drug concentration of a regimen at time t. For the purposes of this query, we will instead stop scaling by the volume of distribution and consider $F(t)$ to be the total drug in the body at time t. The the total amount of antibiotic administered in the first dose of a periodic regimen $F_1(t)$ is just A_0, the initial drug amount. This means the CC regimen with the same total antibiotic amount is given by

$$F_2(t) = D_C := \frac{A_0}{T}$$

where T is the treatment length (the length of one dose of the periodic regimen). Notice that the quantity D_C does not depend on the half-life of the antibiotic. On the other hand, when we begin comparing the performance of both regimens, we will consider

$$\begin{aligned} B_1(T) - B_2(T) &= \int_0^T \left(R\left(F_1(t)\right)\right) dt - \int_0^T \left(R\left(F_2(t)\right)\right) dt \\ &= \int_0^T R\left(A_0 e^{-\lambda t}\right) dt - \int_0^T R\left(\frac{A_0}{T}\right) dt \end{aligned}$$

which *does* depend non-trivially on the half-life t_{half} since $\lambda = \ln(2)/t_{half}$ (where R is the dose response curve). A cursory exploration of the decay model in this context with a

Parameter	Regimen 1	Regimen 2	Regimen 3	Regimen 4
Half-life (h)	4			
Volume of distribution (L)	210			
Periodic dose size (mg)	100	1	30	2.5
Periodic max conc. (mg/L)	0.48	0.0048	0.14	0.012
Periodic min conc. (mg/L)	0.17	0.0006	0.0022	0.0015
Dose period (h)	6	12	24	12
AUC ($mg \cdot h/L$)	1.78	0.024	0.81	0.06
Constant conc. (mg/L)	0.3	0.002	0.034	0.005
$\psi_{\max}$ (h^{-1})	0.88			
$\psi_{\min}$ (h^{-1})	-6.5			
MIC (mg/L)	0.02			
n	1.1			
$\tilde{D}$ (mg/L)	0.0077			
Periodic bac. end value	4.46×10^{-10}	1.48×10^{6}	1.97×10^{-2}	3.00×10^{5}
Constant bac. end value	2.34×10^{-10}	1.50×10^{6}	1.67×10^{-4}	3.04×10^{5}
Which regimen performed better?	Continuous	Periodic	Continuous	Periodic

Table D2: Four ciprofloxacin regimens and the bacteria population value at the end of each treatment interval. Constant concentration is calculated as the AUC of the periodic dose divided by the dose period. Half-life from [2], volume of distribution from [1], PD parameters from [3]. End bacteria population values are given as a percentage of the initial bacteria population

linear dose response curve $R(x) = R_0 + R_1 x$ shows that if $t_{half} > \ln(2)$, then the periodic regimen will always perform better, but if $t_{half} < \ln(2)$, then some relationship between λ and T will determine the regimen which performs better.

We leave a more thorough investigation of holding the total antibiotics administered constant to future work, however it should be noted that this cursory exploration uses the same assumptions on the CC regimen that we used in the rest of this paper, namely that the drug concentration is constant and does not accumulate or decay. When trying to hold the total antibiotic constant instead, we may need to consider the accumulation or decay of the drug in the CC regimen. We did not do this for the rest of the work in this paper because we assumed the concentration in the target tissue was being held constant by an external force such as an IV, disregarding the total amount of antibiotic used. A future investigation should be sure to evaluate appropriate assumptions for holding constant the total antibiotic amount.

Parameter	Reg. 1	Reg. 2	Reg. 3	Reg. 4	Reg. 5
Half-life (h)	2.5				
Volume of dist. (L)	53.2				
Per. dose size (mg)	7000	3100	3000	1400	500
Per. max conc. (mg/L)	131.58	58.27	56.39	26.32	9.40
Per. min conc. (mg/L)	24.93	2.09	2.02	0.94	0.34
Dose period (h)	6	12	12	12	12
AUC (mg $\cdot$ h/L)	384.66	202.62	196.09	91.51	32.68
Cont. conc. (mg/L)	64.11	16.89	16.34	7.63	2.72
$\psi_{\max}$ (h^{-1})	0.7				
$\psi_{\min}$ (h^{-1})	-4.3				
MIC (mg/L)	12.0				
n	2.5				
$\tilde{D}$ (mg/L)	17.67				
Per. end bac. value	7.93×10^{-8}	4.12×10^{-2}	6.98×10^{-2}	1.73×10^{3}	2.41×10^{5}
Cont. end bac. value	8.04×10^{-9}	2.76×10^{-2}	7.25×10^{-2}	2.24×10^{4}	3.50×10^{5}
Which reg. performed better?	Continuous	Continuous	Periodic	Periodic	Periodic

Table D3: Five rifampin regimens and the bacteria population value at the end of each treatment interval. Constant concentration is calculated as the AUC of the periodic dose divided by the dose period. Half-life from [4], volume of distribution from [5], Regoes parameters from [3]. End bacteria values are given as a percentage of the initial bacteria population.

F Code for Figures and Numerical Demonstrations

The code used to generate Figure 1 as well as the numerical results in this paper is available in the following Github repository:
https://github.com/leahchilders/GeneralAntibioticDosing. The code is written in a Jupyter Notebook using Python 3.12.8 and uses the SciPy library for numerical integration. The code is designed to be user-friendly and can be used to analyze any antibiotic data with a selection of PD models. The user can choose a PD model and input their own PK and PD parameters. The code outputs, among other things, the value of $\tilde{D}$, an analytic and numerical analysis of the concavity of the Hill function as performed in this paper, and plots of the dose response curve, the treatment regimens, and the bacteria population curves. The code to reproduce Figure 1 is also provided in a separate Jupyter Notebook.

References

[1] Evan J Begg, Richard A Robson, Darren A Saunders, Garry G Graham, Rona C Buttimore, Alister M Neill, and G Ian Town. The pharmacokinetics of oral fleroxacin and ciprofloxacin in plasma and sputum during acute and chronic dosing. *British Journal of Clinical Pharmacology*, 49(1):32–38, 2000.

[2] Bayer HealthCare Pharmaceuticals Inc. CIPRO- ciprofloxacin hydrochloride tablet, film coated. `https://dailymed.nlm.nih.gov/dailymed/fda/fdaDrugXsl.cfm?setid=888dc7f9-ad9c-4c00-8d50-8ddfd9bd27c0&type=display`, 01 2023.

[3] Roland Regoes, Camilla Wiuff, Renata Zappala, Kim Garner, Fernando Baquero, and Bruce Levin. Pharmacodynamic Functions: a Multiparameter Approach to the Design of Antibiotic Treatment Regimens. *Antimicrobial Agents and Chemotherapy*, 48:3670–6, 10 2004.

[4] Jakko Van Ingen, Rob E Aarnoutse, Peter R Donald, Andreas H Diacon, Rodney Dawson, Georgette Plemper van Balen, Stephen H Gillespie, and Martin J Boeree. Why do we use 600 mg of rifampicin in tuberculosis treatment? *Clinical Infectious Diseases*, 52(9):e194–e199, 2011.

[5] Justin J Wilkins, Radojka M Savic, Mats O Karlsson, Grant Langdon, Helen McIlleron, Goonaseelan Pillai, Peter J Smith, and Ulrika SH Simonsson. Population pharmacokinetics of rifampin in pulmonary tuberculosis patients, including a semimechanistic model to describe variable absorption. *Antimicrobial Agents and Chemotherapy*, 52(6):2138–2148, 2008.
